# Diagnosis of Bovine Respiratory Disease in feedlot cattle using blood ^1^H NMR metabolomics

**DOI:** 10.1038/s41598-019-56809-w

**Published:** 2020-01-10

**Authors:** C. Blakebrough-Hall, A. Dona, M. J. D’occhio, J. McMeniman, L. A González

**Affiliations:** 1 School of Life and Environmental Sciences, Faculty of Science, University of Sydney, Camden, 2570 NSW Australia; 2 Kolling Institute of Medical Research, Faculty of Medicine and Health, University of Sydney, St Leonards, 2065 NSW Australia; 3 Meat and Livestock Australia, Brisbane, 4006 QLD Australia; 4 Sydney Institute of Agriculture, University of Sydney, Biomedical Building, Australian Technology Park, 2015 NSW Australia

**Keywords:** Biochemistry, Metabolomics

## Abstract

Current diagnosis methods for Bovine Respiratory Disease (BRD) in feedlots have a low diagnostic accuracy. The current study aimed to search for blood biomarkers of BRD using ^1^H NMR metabolomics and determine their accuracy in diagnosing BRD. Animals with visual signs of BRD (n = 149) and visually healthy (non-BRD; n = 148) were sampled for blood metabolomics analysis. Lung lesions indicative of BRD were scored at slaughter. Non-targeted ^1^H NMR metabolomics was used to develop predictive algorithms for disease classification using classification and regression trees. In the absence of a gold standard for BRD diagnosis, six reference diagnosis methods were used to define an animal as BRD or non-BRD. Sensitivity (Se) and specificity (Sp) were used to estimate diagnostic accuracy (Acc). Blood metabolomics demonstrated a high accuracy at diagnosing BRD when using visual signs of BRD (Acc = 0.85), however was less accurate at diagnosing BRD using rectal temperature (Acc = 0.65), lung auscultation score (Acc = 0.61) and lung lesions at slaughter as reference diagnosis methods (Acc = 0.71). Phenylalanine, lactate, hydroxybutyrate, tyrosine, citrate and leucine were identified as metabolites of importance in classifying animals as BRD or non-BRD. The blood metabolome classified BRD and non-BRD animals with high accuracy and shows potential for use as a BRD diagnosis tool.

## Introduction

Bovine respiratory disease (BRD) is a multifactorial disease of welfare and economic significance to the feedlot industry globally. Bovine respiratory disease results from a combination of environmental and physiological stressors prior to and upon feedlot entry such as transportation, mixing of unfamiliar animals, and exposure to viral and bacterial agents^1^. Approximately 60–90% of the morbidity and mortality that occurs in feedlots has been attributed to BRD^2,3^. The complex nature of BRD makes establishing a universal ‘gold standard’ for BRD problematic^4^. Current diagnosis methods rely on subjective visual signs of illness, often combined with rectal temperature or lung auscultation to trigger antimicrobial treatment protocols^5^. These diagnosis methods have varying accuracy in diagnosing BRD and the exploration of alternative diagnosis methods is warranted^6–8^.

Metabolomics monitors alterations in the concentration of small metabolites in tissues and biofluids that include lipids, amino acids, vitamins and sugars^9–11^. These metabolites can provide an insight into the response to disease and can be used as biomarkers to indicate the presence of disease^12,13^. Metabolite biomarkers are routinely used in humans to screen for over 30 different disorders including diabetes and heart disease, where metabolic profiling has shown high accuracy for disease detection^14,15^. More recently, many advances in the field of NMR-based metabolomics have been made^16^. Recent improvements include enhanced probe design^17^, higher field magnets and reduction in equipment size^16^, as well as improved methods of identification and quantification^18–20^. These new developments have allowed for enhanced detection of lower concentration metabolites, increased speed of processing, simplification of the complexity of biofluids and more complete spectral assignments. New NMR techniques such as selective optimized flip angle short transient (SOFAST) heteronuclear multiple quantum correlation (HMQC)^21^ and Correlation Spectroscopy (COSY)^22^ have decreased the processing time per sample and succeeded in overcoming issues with overlapping metabolite signals, allowing for proper identification and quantification of metabolites.

Recently, metabolomics has improved the diagnosis of pneumonia and respiratory-related conditions in humans, with metabolites such as L-histidine, glutamic acid and allantoin identified as biomarkers related to the host response to infection^14,23,24^. In cattle, metabolomics has been used to predict production traits such as residual feed intake and average daily gain^25^, as well as diagnosing metabolic and reproductive disorders such as ketosis and metritis^26–28^. To date there has been minimal research on metabolomics as a diagnosis tool for infectious diseases in cattle such as BRD. In the few published studies, between five and twelve metabolites have differentiated healthy and BRD-affected calves^29,30^. A common limitation with studies on biomarker discovery for disease diagnosis in cattle are the small sample sizes of less than 50 animals, which do not allow the validation necessary to ensure reproducibility of the results^9,26,31^.

The objective of the present study was to use the blood metabolome to classify animals as BRD or non-BRD, and to search for blood biomarkers for BRD in feedlot cattle. It was hypothesized that the blood metabolome would contain biomarkers that could be used to classify animals as BRD or non-BRD using the most commonly used BRD diagnosis methods. Gaps in the literature were addressed by using a large sample size and training and validation datasets to ensure reproducibility of the models with future datasets.

## Materials and Methods

The study had approval from the Animal Ethics Committee of Research Integrity and Ethics Administration, The University of Sydney (Approval # 1118). All methods were carried out in accordance with the relevant guidelines and regulations.

### Animals and management

The study was conducted at a commercial cattle feedlot in southern NSW, Australia. Four pens of mixed-breed castrated male cattle (n = 898) were monitored from feedlot entry to slaughter. Animals were sourced from multiple locations and were either purchased at saleyards (n = 788) or direct consignment from cattle properties (n = 110) to enhance the robustness and generalisation of the models to diagnose BRD. Cattle breeds were Angus (n = 187), Angus cross (Hereford x Angus, n = 156), *Bos indicus* cross (n = 29), British cross (British breed mix, less than 75% Angus, n = 82), European (Simmental, Charolais or Limousin, n = 123), Hereford (n = 226), Murray Grey (n = 59) and Shorthorn (n = 36). Cattle entered the feedlot at approximately 1 to 2 years of age. Processing of animals at feedlot entry was staggered over a four-week period in late summer and early autumn. At feedlot entry, animals had initial live weight recorded (mean induction weight was 432 ± SD 51 kg) and were administered standard feedlot treatments upon entry. The treatments included a hormonal growth promotant implant (Revalor S; Coopers Animal Health, NSW, Australia), vaccination for respiratory diseases caused by *Mannheimia haemolytica* (Bovilis MH, Coopers Animal Health, NSW, Australia), live intranasal vaccine for Infectious Bovine Rhinotracheitis (IBR) (Rhinogard, Zoetis Animal Health, New Jersey, USA), 5-in-1 vaccination for clostridial diseases (Tasvax 5 in 1, Coopers Animal Health, NSW, Australia) and an antiparasitic injection (Bomectin, Bayer, Leverkusen, Germany). Following feedlot entry, animals were sent to four production pens for the remainder of their time on feed. Pen 1 housed 300 animals, pen 2 housed 266 animals, pen 3 housed 91 animals and pen 4 housed 241 animals. Animals were fed to allow for ad-libitum feed consumption and were transitioned through three starter rations to a steam-flaked barley-based finisher diet over an 18-day period. The finisher diet contained 71.60% steam-flaked barley, 11.00% cottonseed, 10% wheat distiller’s syrup, 5.30% liquid finisher supplement, 1.80% cottonseed oil and 0.30% barley straw. Animals remained on this ration until slaughter unless they were sent to hospital pens for disease treatment, in which case they received a high roughage (lucerne and barley hay) steam-flaked barley starter diet.

### Animal slaughter

All animals in the study were monitored from induction to slaughter at an average of 112 days on feed (DoF) and lung abnormalities indicative of BRD incidence were recorded using a previously described lung scoring method^32^, where the percentage of lung consolidation on each lobe was summed to form a total percentage of lung consolidation. Pleurisy was recorded using a scoring system of 0 to 3 (Table 1). The use of the term pleuritic tags refers to the adhesion of the lung to the rib cage by fibrous tags.

**Table 1:** Pleurisy scoring system to determine the incidence of Bovine Respiratory Disease in feedlot cattle at slaughter.

Pleurisy Score	Description
0	No pleurisy or pleuritic tags evident on the lungs
1	Tags between lobes or small pleuritic tags on the lung surface
2	Significant pleuritic tags on the lung surface or small pieces of lung adhered to the thoracic wall or significant tags on the lung margins (fringing) or between lobes that could not be broken apart by the inspector
3	All the lung adhered to the thoracic wall with no lung present on the offal table for scoring

### Sampling and clinical measures

Animals were checked daily by highly trained feedlot staff for visual signs of BRD, starting from Day 1 of the study (the day after the first pen of animals entered the feedlot). Animals were scored for signs of BRD in the pen by staff using a modified version of the Wisconsin calf scoring chart^33^. The modified scoring system included assessment of seven visual symptoms: lethargy, head carriage, laboured breathing, cough, nasal discharge, ocular discharge, and rumen fill (Table 2). Animals with visual signs of BRD (n = 149) were removed from their pen and taken for blood sampling and clinical data collection. In order to be diagnosed with BRD based on visual symptoms, animals had to have one or more of the visual symptoms specific to BRD (nasal or ocular discharge, or laboured breathing or cough). This reduced the possibility of mis-diagnosing an animal with BRD. An equivalent number of visually healthy control animals (non-BRD; n = 148) with no visual signs of BRD were removed from the same pen each day and taken for blood sampling and clinical data collection along with the visually sick animals. Animals were between 2 and 42 DoF when they developed visual signs of BRD and were removed from their home pens for clinical assessment, blood sampling for metabolomics and treatment for BRD if required. Data recorded at this time included date, visual identification number, electronic identification number, pen, live weight, rectal temperature and lung auscultation score for both visually sick and visually healthy animals. Rectal temperature was collected using a GLA M750 thermometer (GLA Agricultural Electronics, CA, USA) fitted with a 10 cm probe and inserted for 8 to 15 seconds until maximum temperature was reached. Lung auscultation score was recorded using a Whisper Computer Assisted Lung Auscultation system (Geissler Corporation, MN, USA). The diaphragm of an electronic stethoscope was held over the 5^th^ intercostal space of the right thoracic wall, approximately 10 cm posterior to the elbow, and lung sounds recorded for 8 seconds. Recorded lung sounds were then transmitted wirelessly to a computer containing software to analyse the lung sounds. The Whisper program classifies lung sounds into scores from 1 to 5 (1 = normal, 2 = mild acute, 3 = moderate acute, 4 = severe acute, 5 = chronic). Blood samples for metabolomics analysis were collected from the coccygeal vein of each animal using a 10 ml lithium heparin BD Vacutainer. Samples were placed on ice until centrifugation (2500 × g, 20 min) within one hour of collection. The plasma was transferred to 1.5 ml Eppendorf safe-lock micro test tubes and frozen at −20 °C for a maximum of one month until all sampling was completed and then sent to the laboratory for storage at −80 °C for 5 months prior to analysis.

**Table 2:** Modified calf scoring chart used to assess seven visual signs of Bovine Respiratory Disease of animals in their pens.

Score	0	1	2	3
Lethargy	normal, active, alert	mild lethargy	moderate lethargy	profound lethargy, slow movement
Head Carriage	normal head carriage	poll of head level with the withers	poll of head dropping under withers often	sustained low head carriage
Laboured breathing	normal	mild audible signs of laboured breathing	moderate audible signs of laboured breathing	audible signs of laboured breathing
Cough	none	dry non- productive cough	moderate wet productive cough	severe wet productive cough
Nasal discharge	normal serous discharge	small amount of unilateral cloudy discharge	bilateral, cloudy or excessive mucus discharge	copious bilateral mucopurulent discharge
Eye (ocular discharge)	no ocular discharge	small amount of ocular discharge	moderate amount of ocular discharge	heavy ocular discharge
Rumen Fill	normal	slight depression in rumen fill, no anorexia	moderate depression in rumen fill, no anorexia	severe depression in rumen fill, signs of anorexia

### Bovine respiratory disease reference diagnosis methods

Due to the lack of a universal gold standard for diagnosing BRD, six commonly used methods of BRD diagnosis in feedlots were used as reference methods: visual diagnosis (VD), temperature diagnosis (TD), lung auscultation diagnosis (LAD), clinical diagnosis (CD), visual-clinical diagnosis (VCD) and lung lesion diagnosis (LLD) (Table 3). The sample size for the lung lesion diagnosis method was reduced to 270 animals due to 21 mortalities during the trial period, five animals that were not slaughtered with the main cohort due to injury or lameness, and an additional two animals that were treated for BRD once blood sampling had concluded and therefore could not be included in analysis as their metabolomics sample was taken before the animals developed and were treated for BRD, and therefore was not reflective of their BRD status.

**Table 3:** Description of BRD and non-BRD groups for the six Bovine Respiratory Disease reference diagnosis methods used in the study.

Diagnosis method	Non-BRD	BRD
*Visual Diagnosis*	Score of 0 for visual signs	Score >0 for visual signs
*Temperature Diagnosis*	Rectal temperature <40 °C	Rectal temperature ≥40 °C
*Lung Auscultation Diagnosis*	Lung auscultation score 1	Lung auscultation score ≥2
*Clinical Diagnosis*	Rectal temperature <40 °C and lung auscultation score 1	Lung auscultation score >1 or rectal temperature ≥40 °C
*Visual-Clinical Diagnosis*	Visual diagnosis controls regardless of clinical diagnosis	Case for both visual and clinical diagnosis
*Lung Lesion Diagnosis*	Pleurisy score 1 or lung consolidation >10%	Pleurisy score ≥2 and lung consolidation of >10%, or pleurisy score 3 regardless of lung consolidation percentage

### Blood metabolomics analysis

Samples were prepared for metabolic profiling using methods from a previously published protocol^34^. Samples were thawed at room temperature and an aliquot (350 μL) was mixed with 350 μL of aqueous (80% H_2_O:20% D_2_O) phosphate buffer solution including 0.075 M NaH_2_PO_4_, pH = 7.4 (KOH adjusted), 0.1% sodium azide, and 1 mM 3–141 trimethylsilyl-1- [2,2,3,3, −2H4] propionate (TSP) as an internal standard. Samples were then placed on a vortex for 30 sec and centrifuged at 6,000 x g for 10 min. Aliquots of the supernatants (600 uL) for each plasma sample was then transferred into 5 mm NMR tubes (Bruker, SampleJet 5 mm, Billerica MA, USA) for ^1^H NMR analysis.

Samples were analysed with a Bruker Avance III 600 MHz spectrometer equipped with a 5-mm TCI cryoprobe (Bruker, MA, USA). Samples were run under automation mode using a SampleJet with all samples refrigerated at 4 °C until just prior to acquisition. Data was collected at 310 K for a total of 20 minutes. The delay between acquisition of each scan was set to a 10.00 s as the relaxation of some of the larger proteins and lipoproteins required this to reach an equilibrium. The sample change time, lock, shim and time the sample took to reach temperature was approximately 5 minutes, with a total time between samples being changes of approximately 20 minutes. The noesygrrp1d and cpmgpr1d pulse sequences were used to acquire two water suppressed ^1^H NMR spectra (32 scans collected for each experiment)^34^. During presaturation delay (4.0 s), irradiation of the solvent (water) resonance was applied for all spectra and the noesy during the mixing time (0.01 s)^34^. The pulse sequence parameters including the 90° pulse (~12 μs) receiver gain (~100) were optimised for each sample set run. The data were collected with approximately 96,000 (NOESY) or 32,000 (CPMG) real data points and processed with an exponential line broadening of 0.3 Hz prior to Fourier transformation^35^.

Raw spectrums were imported into Matlab (Mathworks, Natick, MA), automatically phased, baseline corrected and referenced to the α-C^1^H glucose doublet occurring at 5.23 ppm^36^ (Supplementary Fig. 1). The water component was truncated from 4.30 to 5.10 ppm to reduce analytical variation (Supplementary Fig. 2). Probabilistic quotient normalization of the spectrums was performed across all samples, where for each of the individual spectra, a series of quotients is generated by the element-wise division of the spectrum by the reference spectrum. Regions containing no or possibly interfering signals are ignored if possible, and the median of the remaining quotients is subsequently used as correction factor^36,37^. The normalized spectrums were then processed using Standard Recoupling of Variables to calculate the start and end points of each component or component^38^. This method automatically selects bins semi intelligently from the spectra with no set bin size^39^. The area under each component was calculated which represents the relative abundance or concentration of each component^36^. Raw spectrums were then imported into Chenomx NMR Suite (Chenomx, Edmonton, Canada) to identify individual metabolites using reference libraries^36^. The relative concentration of metabolites or components were multiplied by 1,000,000 before analysis to reduce the number of decimal places.

### Data processing and statistical analysis

Classification and regression trees^40^ were used to identify potential biomarkers for BRD and to develop models to predict BRD status using the blood metabolome. This technique searches for metabolites that partition the dataset into BRD and non-BRD groups with the highest accuracy or lowest error rate. Models were developed using entropy method to grow the trees^41^, with cost-complexity pruning^40^. The data was partitioned into training (n = 149) and validation (n = 148) data sets, with half the data used for developing the model and half the data used for validating or evaluate the models’ performance on future data sets. The data was randomly partitioned into a training and validation dataset using the MOD function in SAS where every other observation was sent to the validation dataset. All the metabolite components produced by the NMR spectra were added to the model as predictors, with diagnosis method as the dependent variable. Sensitivity, specificity and accuracy (100 minus error rate) was calculated using the number of true positive, true negative, false positive and false negatives. Sensitivity (Se) was calculated as the frequency with which the model correctly identified BRD animals according to each reference diagnosis method (Se = TP/TP + FN), where TP: True Positive, FN: False Negative). Specificity (Sp) was defined as the frequency with which the model correctly identified non-BRD animals according to each reference diagnosis method (Sp = TN/(TN + FP), where TN: True Negative, FP: False Positive). Accuracy (Acc) was calculated as the sum of the proportion of true positive and true negative animals (Acc = (TP + TN)/(TP + TN + FP + FN)). Sensitivity and 1- specificity were plotted against each other to produce an area under the curve (AUC) which determined the overall accuracy of the models. Principal component analysis (PCA) was performed to obtain a score plot and loading plot of the first two principal components which explained the largest proportion of the variation. Only the first 10 components were retained in the model because these yielded eigenvectors greater than 1. All 34 identified metabolites, along with the twelve components of importance as selected in the CART analysis were labelled in the PCA loading plot. Where more than one component in the spectra belonged to the same metabolite, the component with the clearest, unique peak was used, with the exception of citrate which occurs twice in the plot as it was selected twice by the CART analysis as a metabolite of importance with two unique peaks.

Pearson correlation coefficients were calculated to assess the relationship between the relative concentration of 34 identified metabolite components and the six reference diagnosis methods using point biserial correlations after assigning a value of one to BRD animals and zero to non-BRD animals^42^. Where more than one component in the spectra belonged to the same metabolite, the same component identified in the PCA loading plot was used for the correlation analysis. Mixed-effects linear regression models were used to determine the effect of BRD status on the relative concentrations in arbitrary units^29^ of the same 35 identified metabolites for the visual-clinical diagnosis method only, as presenting data for all diagnosis methods was not possible. The same component used in the PCA and correlation analysis was selected where more than one component in the spectra belonged to the same metabolite. The relative concentrations presented are therefore the concentration of one selected component for each metabolite rather than being the sum of the concentration of all components belonging to the same metabolite. Diagnosis method and breed were included in the models as fixed effects. Where breed was found to be non-significant (P > 0.05) it was removed from the models. Pen number was included in the models as a random effect.

## Results

### BRD diagnosis using blood metabolomics

**Figure 1:**
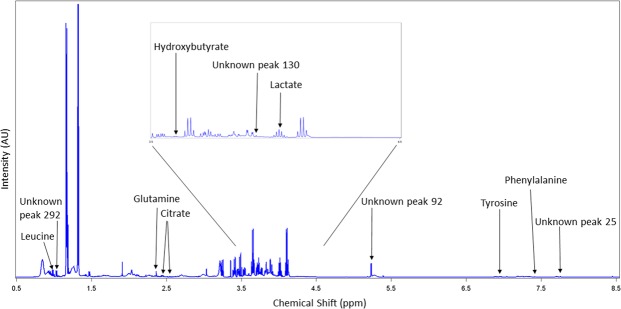
NMR spectrum showing 12 identified metabolites and unknown components that were selected in the classification and regression tree analysis as metabolites of importance in differentiating BRD and non-BRD animals. The region in the box is magnified for clarity.

**Figure 2:**
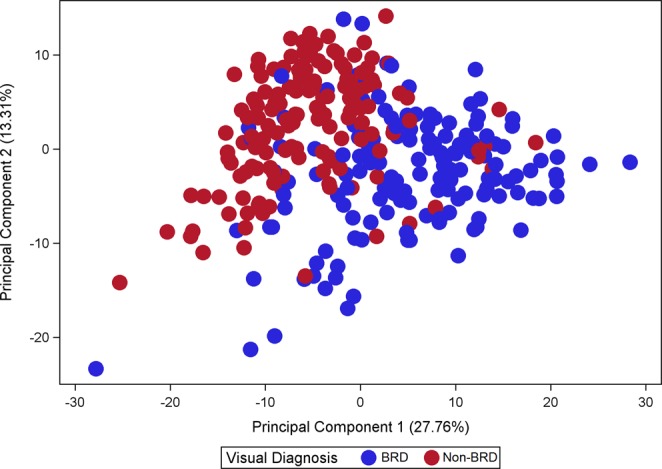
Principal Component Analysis score plot showing the discrimination between BRD animals (blue dots, n = 149) and non-BRD animals (red dots, n = 148) as defined by the visual diagnosis (VD).

**Figure 3:**
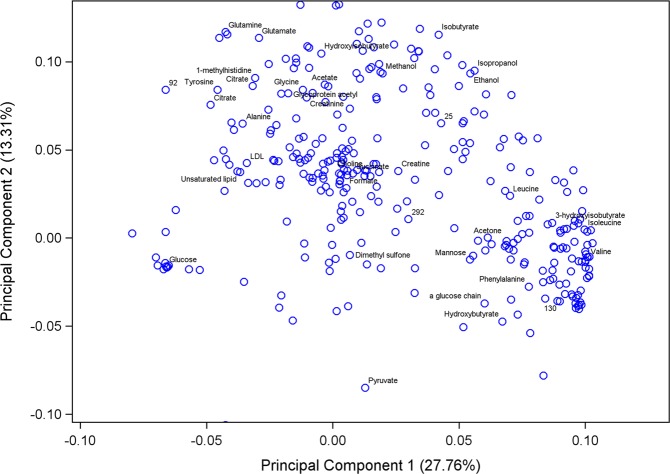
Principal Component Analysis loading plot showing the correlation between metabolites and principal components 1 and 2. All 34 identified metabolites as well as four unknown components that were selected in the classification and regression tree analysis are displayed in the plot. Citrate appears twice as the CART analysis selected two different citrate peaks as components of importance in differentiating BRD and non-BRD animals for two different diagnosis methods.

The ^1^H NMR spectra resulted in 323 metabolite features after processing using the standard recoupling of variables^39^. A full NMR spectra is shown in Fig. 1 with labelling of the 12 identified metabolites and unknown components selected by the CART analysis. The statistical models used were effective in differentiating between BRD and non-BRD animals. The sensitivity, specificity and accuracy of the models developed to classify an animal as either BRD or non-BRD based on their metabolome profile for each of the six reference BRD diagnosis methods are shown in Table 4. Diagnosing BRD using the blood metabolome displayed high accuracy (0.77 to 0.93) in the training datasets; however, the accuracy decreased in the validation data sets (0.64 to 0.85) for all reference diagnosis methods. This decrease in accuracy between the training and validation datasets was more pronounced for the temperature diagnosis, lung auscultation diagnosis and lung lesion diagnosis. The metabolome profile best predicted BRD when defined using the VD and VCD methods. The VD model showed the highest sensitivity (Se = 0.82), specificity (Sp = 0.87) and accuracy (Acc = 0.85) in the validation data set using only two leaves and one component in the final pruned tree (Table 3). This component was an unidentified singlet at 5.39 ppm, which also showed the strongest negative correlation with VD (r = −0.72; Table 5). The VCD diagnosis method produced a model with the highest accuracy (Acc = 0.93) in the training dataset but a decreased accuracy in the validation data set (Acc = 0.81), with 19% of the observations misclassified. This model selected three components (tyrosine, citrate, and hydroxybutyrate) in the final pruned tree to classify an animal as BRD or non-BRD. Diagnosing BRD as defined by LAD had the lowest accuracy in the validation dataset out of all the diagnosis methods with 36% of animals being misclassified. Using the blood metabolome to diagnose BRD as defined by LLD used 5 metabolite components, with 3 of them not able to be identified. This prediction model yielded high accuracy (Acc = 0.92) for the training dataset, however the accuracy decreased to 0.74 in the validation dataset, with a sensitivity of only 0.38. The PCA score plot of the first two principal components (PC) using the visual diagnosis is displayed in Fig. 2 where PC 1 explained 27.76% of the variation and PC 2 explained 13.31% of the variation. The total variance explained by the 10 PC was 68.74% (data not shown). The PCA loading plot of the first two principal components with all 34 identified metabolites labelled is displayed in Fig. 3. The figure shows the clustering of certain metabolites such as dimethyl sulfone, mannose, α-glucose chain, phenylalanine, hydroxybutyrate, valine and pyruvate which had a positive correlation with PC 1 and a negative correlation with PC 2. Similarly, the metabolites glutamine, glutamate, hydroxyisobutyrate, acetate, glycine, glycoprotein acetyl, creatinine, 1-methylhistidine, citrate, tyrosine, alanine, LDL, unsaturated lipid and unknown component 92 showed a positive correlation with PC 2 but a negative correlation with PC 1.

**Table 4:** Sensitivity and specificity of ^1^H NMR metabolomics models to detect Bovine Respiratory Disease in feedlot cattle defined with six reference diagnosis methods.

Diagnosis method	Dataset	Sensitivity	Specificity	Accuracy	AUC^a^	N leaves^b^	N components^c^	Component Number Metabolite ID
*Visual Diagnosis*	Training	0.81	0.93	0.87	0.87	2	1	92
	Validation	0.82	0.87	0.85	0.85	2	1	Unknown (Singlet at 5.39 ppm)
*Temperature Diagnosis*	Training	0.76	0.88	0.85	0.82	2	1	34
	Validation	0.52	0.77	0.69	0.65	2	1	Phenylalanine
*Lung Auscultation Diagnosis*	Training	0.80	0.73	0.77	0.76	2	1	123
	Validation	0.77	0.45	0.64	0.61	2	1	Lactate
*Clinical Diagnosis*	Training	0.77	0.85	0.79	0.81	2	1	227
	Validation	0.79	0.54	0.70	0.67	2	1	Glutamine
*Visual-Clinical Diagnosis*	Training	0.99	0.88	0.93	0.94	4	3	55, 211, 158
	Validation	0.88	0.74	0.81	0.83	4	3	Tyrosine, Citrate, Hydroxybutyrate
*Lung Lesion Diagnosis*	Training	0.76	0.97	0.92	0.90	6	5	219, 130, 292, 305, 25
	Validation	0.38	0.89	0.74	0.71	6	5	Citrate, Unknown, Unknown, Leucine, Unknown

^a^AUC = Area Under the Curve.

^b^N leaves = number of leaves in final pruned tree.

^c^N components = number of components or metabolite components selected in final pruned tree by the classification tree analysis.

**Table 5:** Pearson correlation coefficients between the relative concentration of 34 identified plasma metabolites and Bovine Respiratory Disease defined through six reference diagnosis methods.

Metabolite	Visual	Temperature	Lung Auscultation	Clinical Diagnosis	Visual-Clinical	Lung Lesions
1-methyl-histidine	−0.42^+^	−0.28^+^	−0.28^+^	−0.35^+^	−0.39^+^	−0.20^***^
3-hydroxyisobutyrate	0.45^+^	0.18^**^	0.19^***^	0.25^+^	0.44^+^	0.20^***^
Acetate	−0.28^+^	−0.28^+^	−0.23^+^	−0.25^+^	−0.26^+^	−0.06
Acetone	0.42^+^	0.10	0.21^***^	0.27^+^	0.41^+^	0.18^**^
Alanine	−0.43^+^	−0.23^+^	−0.22^+^	−0.30^+^	−0.41^+^	−0.25^+^
Choline	−0.01	0.02	−0.02	0.01	0.01	−0.13^*^
Citrate	−0.63^+^	−0.35^+^	−0.33^+^	−0.38^+^	−0.61^+^	−0.36^+^
Creatine	0.11	0.17^**^	0.11	0.12^*^	0.14^*^	−0.12^*^
Creatinine	0.49^+^	0.31^+^	0.23^+^	0.31^+^	0.48^+^	0.24^+^
Dimethyl sulfone	0.03	−0.02	−0.03	0.07	0.03	−0.05
Ethanol	0.23^+^	0.06	0.01	0.04	0.23^+^	0.12^*^
Formate	0.04	−0.05	−0.02	0.03	0.06	−0.02
Glucose	−0.41^+^	−0.32^+^	−0.19^***^	−0.29^+^	−0.40^+^	−0.09
Glutamate	−0.59^+^	−0.46^+^	−0.42^+^	−0.48^+^	−0.57^+^	−0.27^+^
Glutamine	−0.63^+^	−0.48^+^	−0.43^+^	−0.51^+^	−0.61^+^	−0.29^+^
Glycine	−0.29^+^	−0.28^+^	−0.24^+^	−0.25^+^	−0.28^+^	−0.10
Glycoprotein acetyls	−0.29^+^	−0.16^**^	−0.22^+^	−0.20^***^	−0.28^+^	−0.12^*^
Hydroxybutyrate	0.64^+^	0.35^+^	0.39^+^	0.44^+^	0.64^+^	0.33^+^
Hydroxyisobutyrate	−0.33^+^	−0.29^+^	−0.22^***^	−0.27^+^	−0.31^+^	−0.14^*^
Isobutyrate	0.15^*^	−0.01	−0.04	−0.02	0.15^**^	0.07
Isoleucine	0.49^+^	0.24^+^	0.23^+^	0.27^+^	0.47^+^	0.27^+^
Isopropanol	0.21^***^	0.05	0.01	0.04	0.21^***^	0.10
Lactate	0.09	0.16^**^	0.16^**^	0.16^**^	0.08	0.01
Leucine	0.47^+^	0.25^+^	0.23^+^	0.26^+^	0.48^+^	0.23^***^
Low Density Lipid	−0.42^+^	−0.22^***^	−0.27^+^	−0.31^+^	−0.42^+^	−0.24^+^
Mannose	0.48^+^	0.36^+^	0.34^+^	0.31^+^	0.48^+^	0.24^+^
Methanol	0.05	−0.12^*^	−0.09	−0.08	0.06	0.03
Phenylalanine	0.49^+^	0.46^+^	0.20^***^	0.30^+^	0.50^+^	0.24^+^
Pyruvate	0.44^+^	0.45^+^	0.32^+^	0.34^+^	0.42^+^	0.12^*^
Succinate	0.09	−0.06	−0.02	0.02	0.10	0.14^*^
Tyrosine	−0.67^+^	−0.49^+^	−0.39^+^	−0.48^+^	−0.65^+^	−0.27^+^
Unsaturated lipid	−0.38^+^	−0.19^***^	−0.25^+^	−0.30^+^	−0.38^+^	−0.23^+^
Valine	−0.48^+^	−0.36^+^	−0.30^+^	−0.34^+^	−0.46^+^	−0.26^+^
α glucose chain	0.66^+^	0.49^+^	0.35^+^	0.38^+^	0.64^+^	0.32^+^

### Metabolite identification and correlation to BRD status

From the 323 spectral components, 34 unique metabolites of bovine plasma were identified using Chenomx software. Pearson correlation coefficients between the relative concentrations of the 34 identified metabolites and BRD status according to the six reference diagnosis methods are shown in Table 5. The strongest correlations between the relative concentrations of metabolites and BRD status were found using the VD and VCD methods, whereas the weakest correlations were found using the TD, LAD, CD and LLD methods. There were metabolites that showed positive correlations with BRD status and others that showed negative correlations. Most metabolites were consistent in the direction of correlation across diagnosis methods. Using the VCD diagnosis definition, BRD status was positively correlated with α-glucose chain (r = 0.64), hydroxybutyrate (r = 0.64) and phenylalanine (r = 0.50; P < 0.001), and negatively correlated with tyrosine (r = −0.65), glutamine (r = −0.61), citrate (r = −0.61), and glutamate (r = −0.57; P < 0.001). Correlation values ranged from −0.67 to 0.66. Some metabolites were significantly correlated to BRD status for all diagnosis methods (1-methyl-histidine, tyrosine, glutamine, citrate, glutamate, valine, LDL (low density lipid), alanine, hydroxyisobutyrate, unsaturated lipid, glycoprotein acetyl, pyruvate, 3-hydroxyisobutyrate, isoleucine, leucine, mannose, phenylalanine, hydroxybutyrate and α-glucose chain). Other metabolites were significantly correlated to BRD status for only some of the diagnosis methods (glucose, glycine, acetate, creatinine, choline, methanol, lactate, succinate, creatine, isobutyrate, isopropanol, ethanol, acetone). Out of these, glucose, glycine and acetate were significantly correlated to BRD for all diagnosis methods except for the lung lesion diagnosis. Only two metabolites (dimethyl sulfone and formate) were not significantly correlated to BRD for any of the diagnosis methods.

### Metabolite concentrations in BRD and non-BRD animals

The correlation analysis results were also reflected in marked differences in the relative concentration of metabolites between BRD and non-BRD animals using the VCD (Table 6). The relative concentrations of metabolites were statistically different between BRD and non-BRD animals for all metabolites except choline, dimethyl-sulfone, formate, lactate, methanol and succinate. Animals with BRD had higher concentrations of 3-hydroxyisobutyrate, α-glucose chain, acetone, creatine, ethanol, hydroxybutyrate, isobutyrate, isoleucine, isopropanol, leucine, mannose, phenylalanine and pyruvate compared to healthy control animals. Animals with BRD had lower concentrations of 1-methyl-histidine, acetate, alanine, citrate, creatinine, glucose, glutamate, glutamine, glycine, glycoprotein acetyl, hydroxyisobutyrate, LDL, tyrosine, unsaturated lipid and valine compared to non-BRD animals.

**Table 6:** Least squares means (±SE) of the relative concentrations in arbitrary units of 34 identified metabolites for the visual-clinical diagnosis method only. The metabolites in bold were not significantly different between BRD and non-BRD animals (P > 0.05).

Metabolite	Visual-Clinical Diagnosis		
	Non-BRD n = 152	BRD n = 142	P-Value
1-methyl-histidine	1.30 ± 0.035	1.10 ± 0.035	<0.001
3-hydroxyisobutyrate	10.41 ± 0.262	12.74 ± 0.271	<0.001
α glucose chain	0.79 ± 0.023	1.26 ± 0.024	<0.001
Acetate	9.76 ± 0.382	7.72 ± 0.397	<0.001
Acetone	2.22 ± 0.082	3.13 ± 0.086	<0.001
Alanine	7.59 ± 0.141	6.68 ± 0.144	<0.001
**Choline**	**5.86** ± **0.098**	**5.87** ± **0.102**	**0.941**
Citrate	2.15 ± 0.054	1.63 ± 0.055	<0.001
Creatine	10.87 ± 0.214	11.60 ± 0.225	0.020
Creatinine	4.11 ± 0.104	4.90 ± 0.107	<0.001
**Dimethyl sulfone**	**2.58** ± **0.124**	**2.67** ± **0.131**	**0.602**
Ethanol	27.13 ± 0.220	28.43 ± 0.232	<0.001
**Formate**	**0.47** ± **0.024**	**0.50** ± **0.025**	**0.330**
Glucose	23.13 ± 0.216	20.82 ± 0.228	<0.001
Glutamate	4.36 ± 0.055	3.60 ± 0.057	<0.001
Glutamine	2.83 ± 0.042	2.15 ± 0.043	<0.001
Glycine	14.54 ± 0.238	12.97 ± 0.249	<0.001
Glycoprotein acetyls	14.72 ± 0.217	13.18 ± 0.228	<0.001
Hydroxybutyrate	5.78 ± 0.079	7.10 ± 0.082	<0.001
Hydroxyisobutyrate	2.37 ± 0.033	2.10 ± 0.035	<0.001
Isobutyrate	181.78 ± 1.811	188.20 ± 1.899	0.009
Isoleucine	12.50 ± 0.291	14.65 ± 0.297	<0.001
Isopropanol	13.19 ± 0.120	13.84 ± 0.126	<0.001
**Lactate**	**57.96** ± **2.395**	**62.46** ± **2.517**	**0.181**
Leucine	6.31 ± 0.076	7.33 ± 0.080	<0.001
Low Density Lipid	21.40 ± 0.380	17.11 ± 0.400	<0.001
Mannose	0.75 ± 0.020	1.02 ± 0.021	<0.001
**Methanol**	**10.04** ± **0.116**	**10.20** ± **0.122**	**0.342**
Phenylalanine	0.47 ± 0.015	0.62 ± 0.016	<0.001
Pyruvate	5.33 ± 0.107	6.54 ± 0.113	<0.001
**Succinate**	**1.78** ± **0.037**	**1.87** ± **0.039**	**0.091**
Tyrosine	0.67 ± 0.014	0.46 ± 0.015	<0.001
Unsaturated lipid	4.40 ± 0.092	3.46 ± 0.097	<0.001
Valine	4.82 ± 0.114	3.90 ± 0.117	<0.001

## Discussion

The present study evaluated the potential of blood metabolomics to differentiate between BRD and non-BRD animals. There have been few studies on characterizing the metabolome of healthy control animals to identify baseline metabolite concentrations^9^. We used a case-control study to capture the metabolome profile of BRD and non-BRD animals for appropriate model development and evaluation. Classification and regression trees (CART) were used to search for potential biomarkers for BRD and to develop prediction models. This technique was selected for developing the prediction models and for biomarker discovery as it allows for predictive modelling of the data and identification of specific biomarkers, as well as being relatively simple to understand^43^. Using this statistical method ensured that the models generated could be applicable to future datasets.

The models identified 12 metabolite components, or biomarkers, of importance in differentiating BRD and non-BRD animals. From these, eight out of 12 components were identified as seven unique metabolites (phenylalanine, lactate, glutamine, hydroxybutyrate, tyrosine, citrate and leucine). Previous studies searching for biomarkers for BRD in cattle have identified metabolites such as glucose, valine, phosphorous, propionate, acetate, phenylalanine, 2-methylglutarate and phenol as biomarkers for viral BRD infection^29,30,44^. In human pneumonia, six metabolites (uric acid, L-histidine, hypoxanthine, glutamic acid, L-tryptophan and ADP) have been identified as markers related to the host response to infection through energy metabolism and inflammatory and antimicrobial pathways^23^.

The blood metabolome best predicted BRD when using the visual diagnosis method, with 85% of animals correctly classified as either BRD or non-BRD in the validation dataset. This is lower than a previous study which reported an accuracy of 95% when diagnosing BRD calves using a sample size of 50 BRD animals and only 10 non-BRD animals^29^. The greater accuracy of metabolomics to predict BRD reported by these authors could be due to the statistical methods and experimental design they used, using only 10% of the data for validation and the whole spectra as predictors rather than the components or peaks as in our study^29^. The 85% accuracy of the VD model was obtained using only one metabolite component which could not be identified. Interestingly, this unidentified component was also the component with the lowest relative concentration in BRD animals and showed the strongest correlation to BRD status. This is an important finding because it suggests that it is possible to achieve a high accuracy with only one biomarker, making the technique potentially practical and simple for applications under commercial conditions. Results of the PCA analysis using the visual diagnosis method support the findings of the CART analysis, showing good separation between BRD and non-BRD animals with a large proportion of the variance explained. The PCA analysis showed clustering of certain metabolites associated with different diagnosis methods. Hydroxybutyrate and phenylalanine were clustered together and these metabolites were also selected by the CART analysis as important in differentiating BRD and non-BRD animals when using rectal temperature and the visual-clinical diagnosis. The clustering of certain metabolites associated with similar methods of BRD diagnosis could indicate these metabolites are involved in related physiological functions or pathways of BRD infection and progression.

Compared to the VD and VCD diagnosis methods, the accuracy of metabolomics to correctly diagnose BRD decreased when using the other four reference diagnosis methods in the present study. This is most likely because blood samples for metabolomics analysis were taken relative to the day animals exhibited obvious visual signs of BRD, however the time of sampling may not have coincided with the maximal change in rectal temperature, abnormal lung sounds or when lesions in the respiratory tract appear. The sequence of events with episodes of BRD include an initial elevation in rectal temperature as a result of the febrile response to an invading pathogen, followed by the development of clinical signs such as depression and ocular and nasal discharge, with the development of abnormal lung sounds occurring up to five days after the initial fever episode^45^. Viral pathogens associated with BRD can take 7 to 9 days before a defence response is activated, whereas bacterial pathogens can cause a more rapid acute phase response through faster action of endotoxin infection^46^. Different metabolites were associated with the six reference diagnosis methods used in our study and this could be related to the stage or nature of the infection, extent of tissue damage or the physiological responses of the animal to the disease. For instance, lactate was selected as a metabolite of importance for the lung auscultation diagnosis. In this instance there may be a reduced oxygen transfer from the lungs to the arterial blood, or elevated oxygen consumption due to increased breathing which would increase the importance of anaerobic pathways and increase lactate levels^47^. These results indicate that the blood metabolome could be useful in differentiating BRD causative agents (viral versus bacterial), stage of infection, or disease severity. However, further research is needed to understand changes in the blood metabolome profile with the onset of BRD and with underlying causative agents. To determine changes in the blood metabolome with the progression of disease and with different BRD causative pathogens, samples would need to be obtained frequently over time, starting before pathogen infection up until slaughter.

The accuracy of the prediction models decreased when applied to the validation datasets, with some models demonstrating a larger decrease than others. The models that used the VD and VCD as reference diagnosis methods displayed a much smaller decrease in accuracy from the training to the validation dataset, indicating they are likely to be more robust on future datasets. The decrease in accuracy from the training to validation dataset is expected as the latter is an independent dataset not used to develop and train the model^48^. Although partially independent, the training and validation datasets used in this study were not completely independent because animals were part of the same experimental study conditions and therefore need to be tested under broader conditions (different feedlots, regions and animals). Despite not being completely independent, the models produced high accuracies considering the complex study design. Animals originated from multiple sources, were of mixed breed and age, had differing live weight and body condition and were sampled on different days in differing environmental conditions. Future work in this area should utilise independent datasets from multiple feedlots to further validate the models developed and evaluate their potential utility as a BRD diagnosis tool.

The NMR spectra obtained resulted in 323 metabolite components, with 106 of these assigned to one of 34 known metabolites of bovine plasma. The strongest correlations of metabolites to BRD status were found using the visual diagnosis and visual-clinical diagnosis. This suggests there is a significant impact of disease status on the blood metabolome. Most identified metabolites (20) showed significant correlations to BRD with all six reference diagnosis methods. Of interest is that many metabolites were significantly correlated to BRD when defined by lung lesions at slaughter. This result has not been described before and suggests that certain metabolites are related to the underlying lung pathology of BRD. The fact most metabolites were consistent in the direction of the correlation across all six diagnosis methods suggests a common pattern and possible common underlying mechanisms associated with these metabolites and BRD status.

All 34 identified metabolites showed statistical differences in relative concentration between BRD and non-BRD animals except for six (choline, dimethyl-sulfone, formate, lactate, methanol and succinate). Animals suffering from BRD had higher concentrations of 3-hydroxyisobutyrate, α-glucose chain, acetone, creatine, creatinine, ethanol, hydroxybutyrate, isobutyrate, isoleucine, isopropanol, leucine, mannose, phenylalanine and pyruvate. From these metabolites, the statistical models selected phenylalanine, leucine and hydroxybutyrate as metabolites of importance in diagnosing BRD. Phenylalanine has previously been shown to be higher in calves with bronchopneumonia^29^ and human patients with pneumonia and sepsis^49,50^. A higher concentration of phenylalanine in diseased patients potentially represents a reduction in its conversion to tyrosine when under oxidative stress as a result of the immune response^51^. This could explain the higher concentrations of phenylalanine and lower concentrations of tyrosine seen in BRD animals in the present study. The elevated levels of leucine, a branched-chain amino acid, could be due to the increased requirements for protein synthesis associated with oxidative stress following inflammation and disease^52^. Hydroxybutyrate, a ketone body that accumulates in the bloodstream under negative energy balance has been shown to increase in many disease states^50,53,54^. Our finding of greater concentrations of hydroxybutyrate and ethanol in BRD animals is contrast to the trends reported by Basoglu *et al*. (2016), who found lower levels of hydroxybutyrate and ethanol in animals suffering from bronchopneumonia. Differences found in the concentrations of metabolites between sick and healthy animals across studies could be due to differences in analytical and statistical techniques, the type of animals used in the study and the possible causative agents involved.

Animals suffering from BRD had lower relative concentrations of 1-methyl-histidine, acetate, alanine, citrate, glucose, glutamate, glutamine, glycine, glycoprotein acetyl, hydroxyisobutyrate, LDL, tyrosine, unsaturated lipid and valine. From these, the models selected glutamine, citrate and tyrosine as metabolites of importance in diagnosing BRD. Lower levels of citrate and tyrosine have previously been observed in human pneumonia cases^55^. The reduction of citrate, an energy substrate and key metabolite in the tricarboxylic acid cycle, could be due to the increased energy expenditure required for immune cell production with the onset of disease and increased glucose oxidation, leading to decreased levels of energy substrates in the blood stream^56^. A previous study demonstrated that citrate and hydroxybutyrate follow opposite trends and are negatively correlated in dairy cows with ketosis and metritis^53^. In agreement with the present study, tyrosine and glutamate were shown to decrease in cattle experimentally infected with lipopolysaccharide (LPS) from the bacteria *Escherichia coli*^57^. Tyrosine and glutamate have also followed a similar pattern in a study on human sepsis biomarkers^50^. The lower levels of acetate, glucose, LDL and valine in animals suffering from BRD are in agreement with previous studies^29,44^. Many of the metabolites we found to be significantly different between BRD and non-BRD animals are in agreement with previous studies, indicating their potential utility in BRD diagnosis. However, further research is warranted to explore the predictive ability and prognostic value of these biomarkers in broader conditions and to determine the stability, or duration which they are detectable.

The present study has demonstrated that ^1^H NMR metabolomics is a feasible approach for the identification of biomarkers of BRD and that biomarkers identified by the statistical models were accurate at classifying animals as BRD or non-BRD. The sample size, diversity of the animals used and the validation of our models increased the robustness and generalisation of our results for future applications. We demonstrated that one to five metabolites could accurately diagnose BRD, indicating their potential for simple and rapid crush-side diagnosis tests. The accuracies obtained using the VD and VCD diagnosis were comparable to, if not higher than many of the currently used BRD diagnosis methods. Furthermore, most metabolites showed a significant correlation to BRD status, indicating a significant impact of disease status on an animal’s metabolite profile. The fact many of the metabolite biomarkers we identified were consistent with other studies confirms the potential utility of metabolomics as a BRD diagnosis tool to aid in the confirmation of BRD following the initial visual diagnosis. Further validation of these biomarkers is needed to explore their potential as a BRD diagnosis tool.

## Supplementary information

Supplementray information.

## Data Availability

Data is available upon request from Luciano Adrian Gonzalez (luciano.gonzalez@sydney.edu.au).
