# Supplementary material for: Diagnosis of Bovine Respiratory Disease in feedlot cattle using blood 1H NMR metabolomics
Source: Sci Rep. 2020 Jan 10;10:115. doi: 10.1038/s41598-019-56809-w (PMC6954258; doi:10.1038/s41598-019-56809-w)

Diagnosis of Bovine Respiratory Disease in feedlot cattle using blood ^{1}H NMR metabolomics

C. Blakebrough-Hall[1*], *A. Dona*[2], *M.J. D'occhio*[1], *J. McMeniman*[3] *and L.A González*[1,4]

[1]School of Life and Environmental Sciences, Faculty of Science, University of Sydney, Camden, 2570, NSW, Australia
[2]Kolling Institute of Medical Research, Faculty of Medicine and Health, University of Sydney, St Leonards, 2065, NSW, Australia
[3]Meat and Livestock Australia, Brisbane, 4006, QLD, Australia
[4] Sydney Institute of Agriculture, University of Sydney, Biomedical Building, Australian Technology Park, 2015, NSW, Australia

Supplementary Information

Supplementary Figure 1. Full spectra before water peak (4.30 to 5.10 ppm) and DSS (4,4-dimethyl-4-silapentane-1-sulfonic acid; -1.00 to 0.52 ppm) truncation. DSS is used for calibration of the NMR.

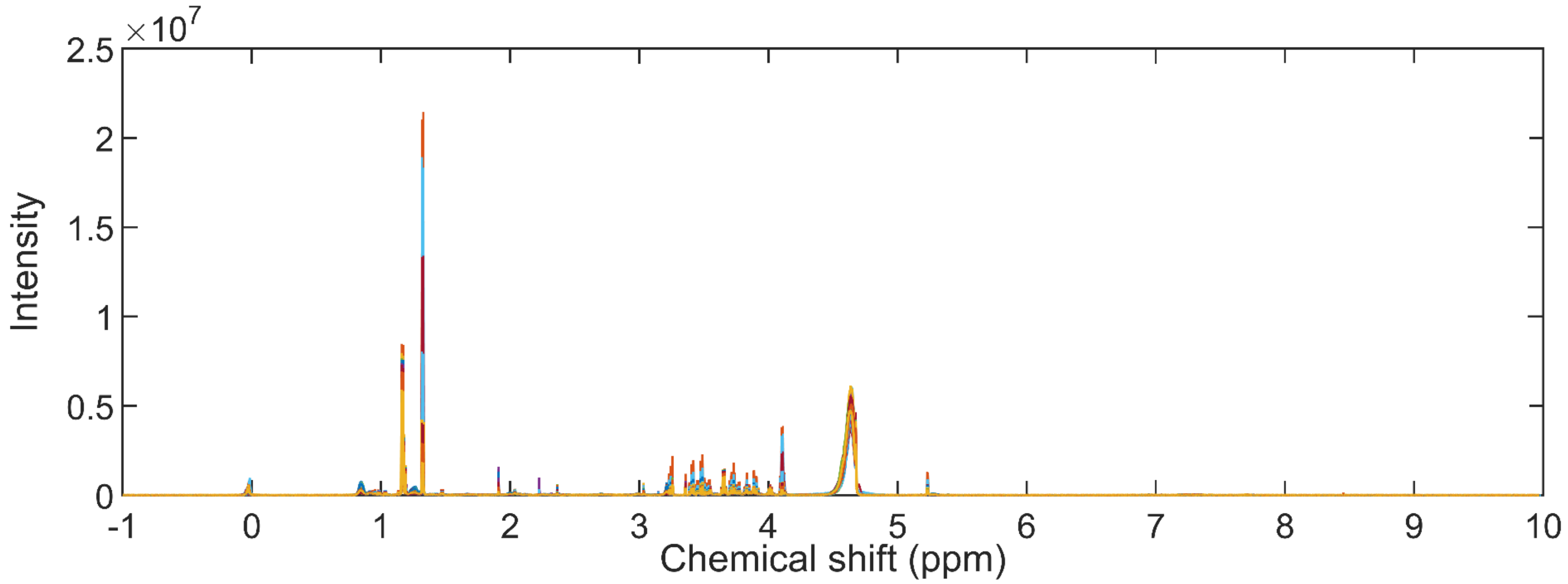

Supplementary Figure 2. Full spectra after water peak (4.30 to 5.10 ppm) and DSS (-1.00 to 0.52 ppm) truncation.

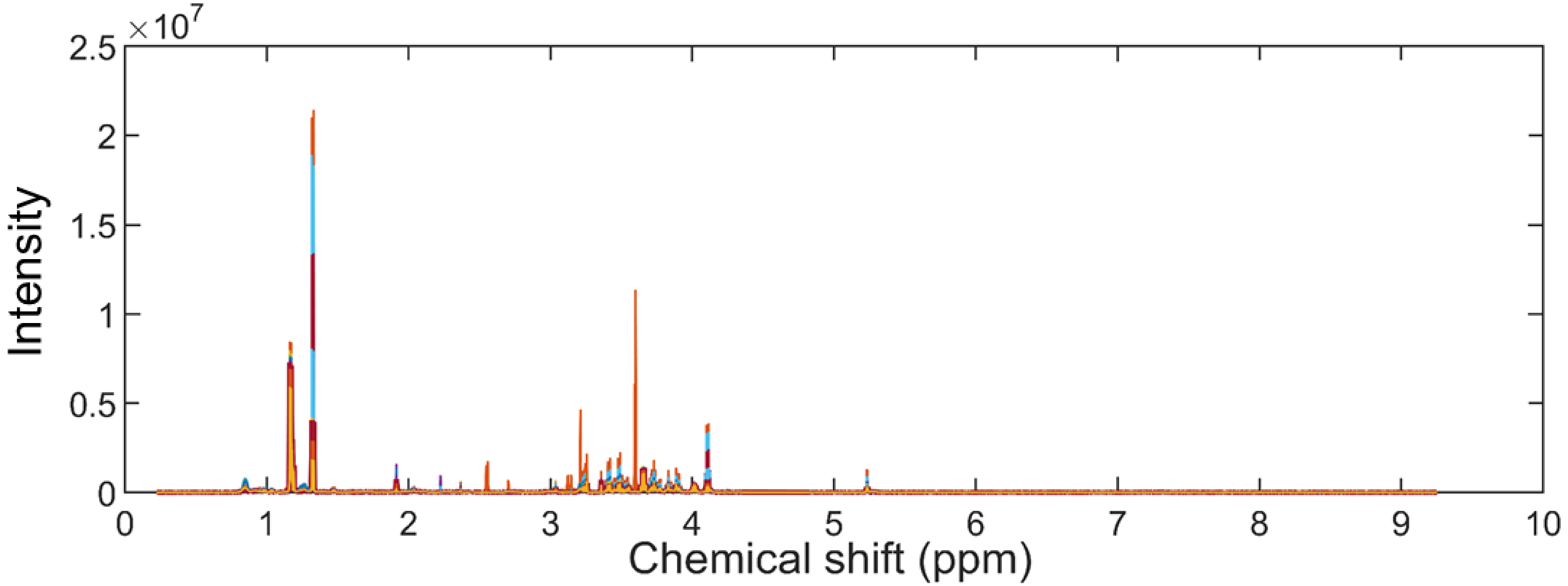

Supplement: Supplementary file 1 — Supplementray information. [file 41598_2019_56809_MOESM1_ESM.pdf]
